# A novel frameshift variant of *PAX3* in a Chinese Yugur family with Waardenburg syndrome type 1

**DOI:** 10.3389/fgene.2025.1679351

**Published:** 2025-10-22

**Authors:** Lupeng Zhan, Baicheng Xu, Dujuan Lin, Ya Wang, Panpan Bian

**Affiliations:** Department of Otolaryngology-Head and Neck Surgery, Lanzhou University Second Hospital, Lanzhou, China

**Keywords:** *PAX*3, Waardenburg syndrome, whole-exome sequencing, de novo mutation, hearing loss

## Abstract

**Introduction:**

Waardenburg syndrome type 1 (WS1) is a rare autosomal dominant disorder characterized by congenital sensorineural hearing loss and facial dysmorphisms. *PAX3* mutations are a known genetic cause. This study investigated a novel *PAX3* mutation in a Chinese Yugur family and assessed long-term auditory outcomes after cochlear implantation.

**Methods and Results:**

Whole-exome and Sanger sequencing were used to identify causative variants, followed by bioinformatic analyses to predict pathogenicity. Auditory and speech rehabilitation were evaluated over a 7-year follow-up. A novel heterozygous frameshift mutation in PAX3 (c.788dup, p.Gln264ThrfsTer5) was identified and confirmed to be *de novo*. Structural modeling indicated disruption of a conserved domain, supporting its pathogenic role. The proband achieved excellent auditory and speech recovery and successfully integrated into mainstream education.

**Discussion:**

This study expands the mutation spectrum of PAX3 and provides evidence supporting the pathogenicity of the c.788dup variant. It also confirms the long-term benefit of cochlear implantation and rehabilitation in WS1-related hearing loss.

## 1 Introduction

Waardenburg syndrome (WS) is recognized as the most common form of syndromic deafness inherited in an autosomal dominant pattern, first documented by the Dutch clinician and geneticist Petrus Johannes Waardenburg (Waardenburg, 1951). Its global prevalence is approximately one in 42,000, accounting for 2%–5% of congenital deafness cases (George et al., 2023; Tawfik et al., 2025). WS displays extensive phenotypic and genetic heterogeneity, typically involving sensorineural hearing impairment, hypertelorism, pigmentary irregularities in hair, skin, and eyes, and, less frequently, musculoskeletal defects. In 1992, Farrer et al. proposed clinical diagnostic criteria based on major and minor criteria (Farrer et al., 1992). Major criteria include: congenital hearing loss, heterochromia iridis, white frontal hair, wide-set eyes (W index >1.95), and a confirmed case among first-degree relatives; minor criteria include: skin vitiligo, fused eyebrows, excessive eyelashes, broad nasal bridge, underdeveloped nasal wings, and abnormal upper lip. A clinical diagnosis of WS1 is established when a patient fulfills either two major criteria or one major criterion in combination with at least two minor criteria. Based on WS1, WS is further classified into four subtypes according to different accompanying symptoms. WS1 is distinguished by an increased interpupillary distance (Guo et al., 2021). WS2 shares all characteristics with WS1 except for the absence of widened interpupillary distance (Han et al., 2025). WS3 presents with skeletal and muscular system abnormalities (such as upper limb deformities) in addition to WS1 (Tekin et al., 2001). WS4 combines the features of WS2 with Hirschsprung’s disease (HD), which involves congenital megacolon and intestinal atresia (Gombojav et al., 2025). Currently, researchers have identified six WS-related genes, including *PAX3*, *MITF*, *SNAI2*, *EDN3*, *EDNRB*, and *SOX10* (Lee et al., 2022). These genes are critical in WS pathogenesis. Among them, *PAX3* is predominantly associated with WS1 and WS3, while other subtypes involve different genetic mutations (Han et al., 2025).

The *PAX3* gene (https://omim.org/entry/606597) codes for a transcription factor classified within the paired box family. Positioned at 2q36.1 on chromosome 2, *PAX3* consists of 10 exons and encodes a protein comprising 479 amino acids. The PAX3 protein contains several evolutionarily conserved regions, including the paired box domain (PD) at the N-terminus, the octapeptide (O), and the paired-type homeodomain (HD), as well as the transcription activation domain (TAD) rich in serine-threonine-proline residues at the C-terminus. The PD and HD domains form the DNA-binding region, which collaboratively recognize and bind to the regulatory sequences of specific target genes, thereby regulating downstream target gene expression (Moore et al., 2025). These domains are crucial for the PAX3 protein’s biological functions. The integrity of the PD and HD domains ensures that PAX3 can regulate the expression of key target genes during embryonic development (Boudjadi et al., 2018). Pathogenic mutations in *PAX3* disrupt its ability to regulate cell proliferation, differentiation, migration, and survival, particularly in cells derived from neural crest cells (NCCs), leading to the onset of WS1 and WS3 symptoms (Matsunaga et al., 2013).

In this study, we report a novel *de novo* heterozygous frameshift mutation (c.788dup, p.Gln264ThrfsTer5) in exon five of *PAX3* in a 17-year-old female from a Chinese Yugur family with WS1. Clinical features included congenital bilateral sensorineural hearing loss, heterochromia iridis, and dystopia canthorum. This variant was not found in her unaffected parents and has not been previously reported. Our findings expand the *PAX3* mutation spectrum and provide insight into the genetic basis of WS1.

## 2 Materials and methods

### 2.1 Clinical and hearing assessment

This study investigated a sporadic deafness family from Zhangye City, Gansu Province, China. Family history and comprehensive otologic and ophthalmologic evaluations were conducted. Audiological assessments included pure-tone audiometry (PTA), auditory brainstem response (ABR), impedance tests, and cranial imaging. Ocular and physical features were examined using fundus imaging, optical coherence tomography, and limb and pigmentation inspections. Hearing loss severity was categorized per WHO standards. Post-cochlear implantation outcomes were assessed through standardized scales such as the Categories of Auditory Performance (CAP), the Speech Intelligibility Rating (SIR), the Meaningful Auditory Integration Scale (MAIS), and the Meaningful Use of Speech Scale (MUSS). The W index was calculated using an established formula incorporating canthal and interpupillary measurements to evaluate dystopia canthorum. This study was approved by the Ethics Committee of the Second Hospital of Lanzhou University (certificate no. 2021A-027), and written informed consent was obtained from all participants.

### 2.2 Sample collection

2 mL of peripheral blood were collected from the patient and both parents using EDTA anticoagulant vacuum tubes. Samples were stored at −80 C and transported under cold-chain conditions to the Beijing Mygenostics Medical Testing Institute. Genomic DNA was extracted using a genomic DNA extraction kit (Mygenostics, Kangwei Century, QIAGEN). DNA concentration and purity were assessed with a Nanodrop 2000 UV spectrophotometer. Only samples with a concentration ≥30 ng/μL and an OD260/280 ratio between 1.7 and 2.0 were used for further analysis.

### 2.3 Whole exome sequencing

WES was performed by the Beijing Mygenostics Medical Testing Institute (Beijing, China). High-quality genomic DNA (1–3 μg) was enzymatically sheared to produce fragments averaging 150 base pairs in length. Using a standard DNA library preparation kit, the sequencing library was built through end-repair, ligation of adapters, and amplification via PCR. Whole-exome enrichment was performed with the P039-Exome probe set developed by Mygenostics, and sequencing was carried out on the Illumina NextSeq 500. Raw sequencing data were cleaned with Cutadapt v1.16 to strip adapter sequences, filter low-quality bases, remove reads with excessive N bases, and exclude those under 40 bp. Reads passing quality control were mapped to the human genome reference sequence (hg19). Post-alignment processing included filtering with SAMtools/BamTools, duplicate removal with GATK MarkDuplicates, and base quality recalibration using GATK BaseRecalibrator. Genetic variants, including single nucleotide polymorphisms (SNPs), insertions and deletions (Indels), and copy number variations (CNVs), were identified using GATK HaplotypeCaller v4.0.8.1. Variant annotation was performed with ANNOVAR. Pathogenicity interpretation followed the ACMG guidelines, integrating population databases such as gnomAD, ClinVar, and HGMD, along with prediction tools like MutationTaster.

### 2.4 Sanger sequencing

To validate the detected variant, Sanger sequencing was conducted. Primer pairs were designed using Primer3 software. The specific sequences were: Forward primer: 5′-CAA​AGT​CCT​AAC​AAT​ATG​CAT​CCC-3′; Reverse primer: 5′-TGC​AGT​CGG​AGA​GAG​AAC​TTG-3′. PCR amplification was carried out using 2X GoldStar Master Mix. The amplification protocol involved an initial heating step at 95 C for 10 min, followed by 36 repetitive cycles of denaturation (94 C, 30 s), annealing (58 C, 30 s), and extension (72 C, 45 s). An additional extension step at 72 C for 5 min was performed to complete the amplification process. PCR amplicons were checked through agarose gel electrophoresis and then isolated using magnetic bead purification. Subsequent sequencing was performed using capillary electrophoresis on an ABI 3130XL Genetic Analyzer. Using Mutation Surveyor software, the sequence data were aligned to the PAX3 reference sequence (NM_181458.4) for variant identification.

### 2.5 Evolutionary conservation analysis

PAX3 protein sequences in FASTA format were obtained from the NCBI database (https://www.ncbi.nlm.nih.gov/) for representative species, including humans, mice, chimpanzees, and rhesus monkeys. The collected sequences were subsequently imported into UGENE software (version 46.0; Unipro UGENE, https://ugene.net/) for comparative analysis. Sequence alignment was conducted utilizing the Clustal W algorithm integrated within UGENE. The conservation of the mutant site, along with flanking upstream and downstream amino acid regions, was then evaluated visually.

### 2.6 Three-dimensional structure modeling

The wild-type PAX3 protein sequence was accessed from the UniProt database (https://www.uniprot.org/).The c.788dup mutation introduced a frameshift, generating a truncated protein product due to the introduction of an early stop codon. Both wild-type and mutant amino acid sequences were prepared in FASTA format and uploaded to the AlphaFold database (https://alphafold.ebi.ac.uk/) for structural prediction using homology modeling techniques. Default parameters were employed to obtain PDB files. The resulting models were visualized and compared using PyMOL software (v2.5, Schrödinger Inc.). The align command was applied to superimpose the wild-type and mutant structures to assess global conformational differences. A “rainbow” color scheme was used to illustrate the sequential regions of the proteins, and three-dimensional images were generated for further analysis.

## 3 Result

### 3.1 Clinical features and rehabilitation

**FIGURE 1:**
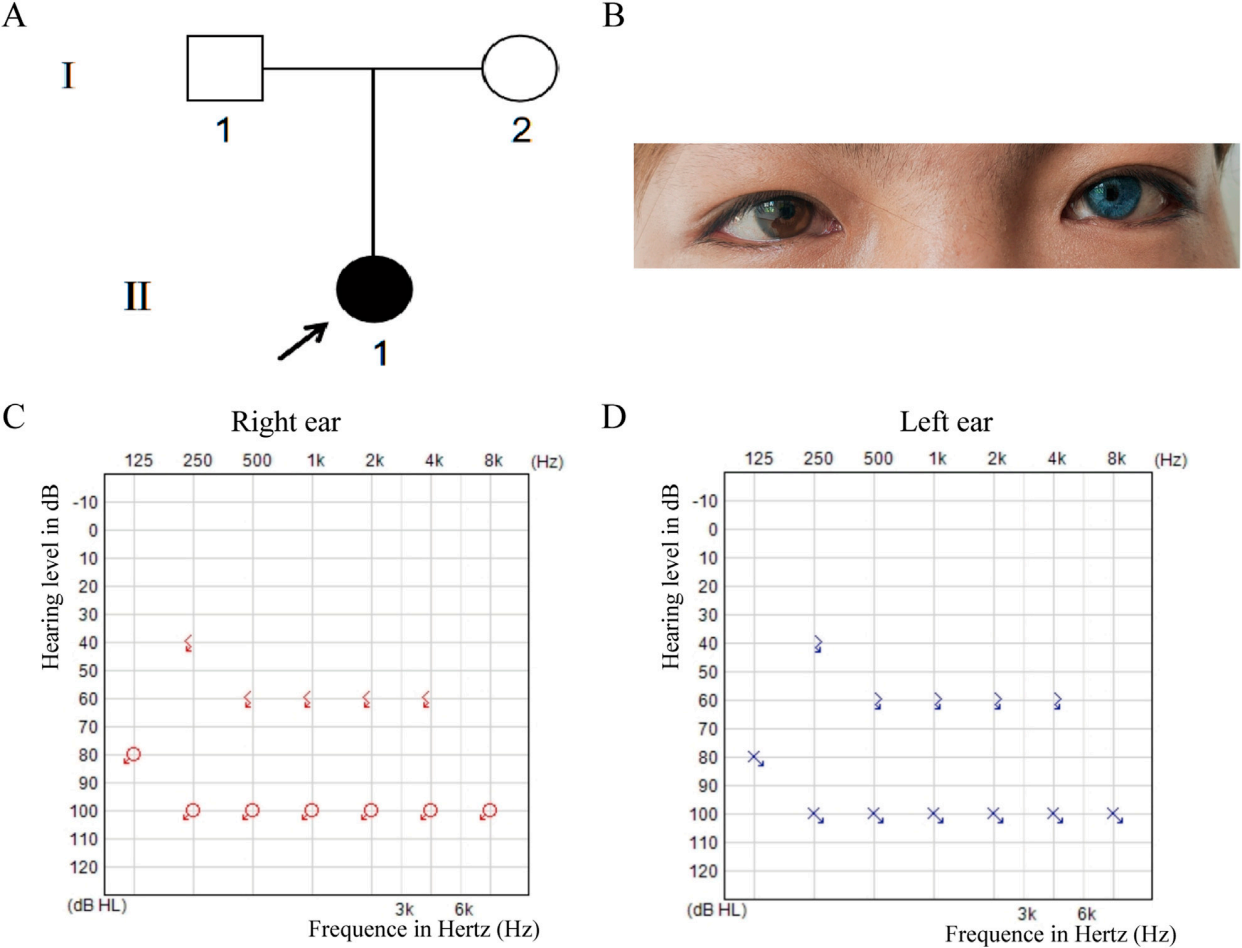
Pedigree, Clinical Features and Hearing Tests. **(A)** Pedigree shows a sporadic case with the proband (II-1, arrow) as the only affected member. **(B)** The proband has iris heterochromia (brown left, blue right), wide-set eyes, and a thick left eyebrow. **(C,D)** Preoperative pure-tone audiometry revealed profound bilateral sensorineural hearing loss at all frequencies (>90 dB HL).

**FIGURE 2:**
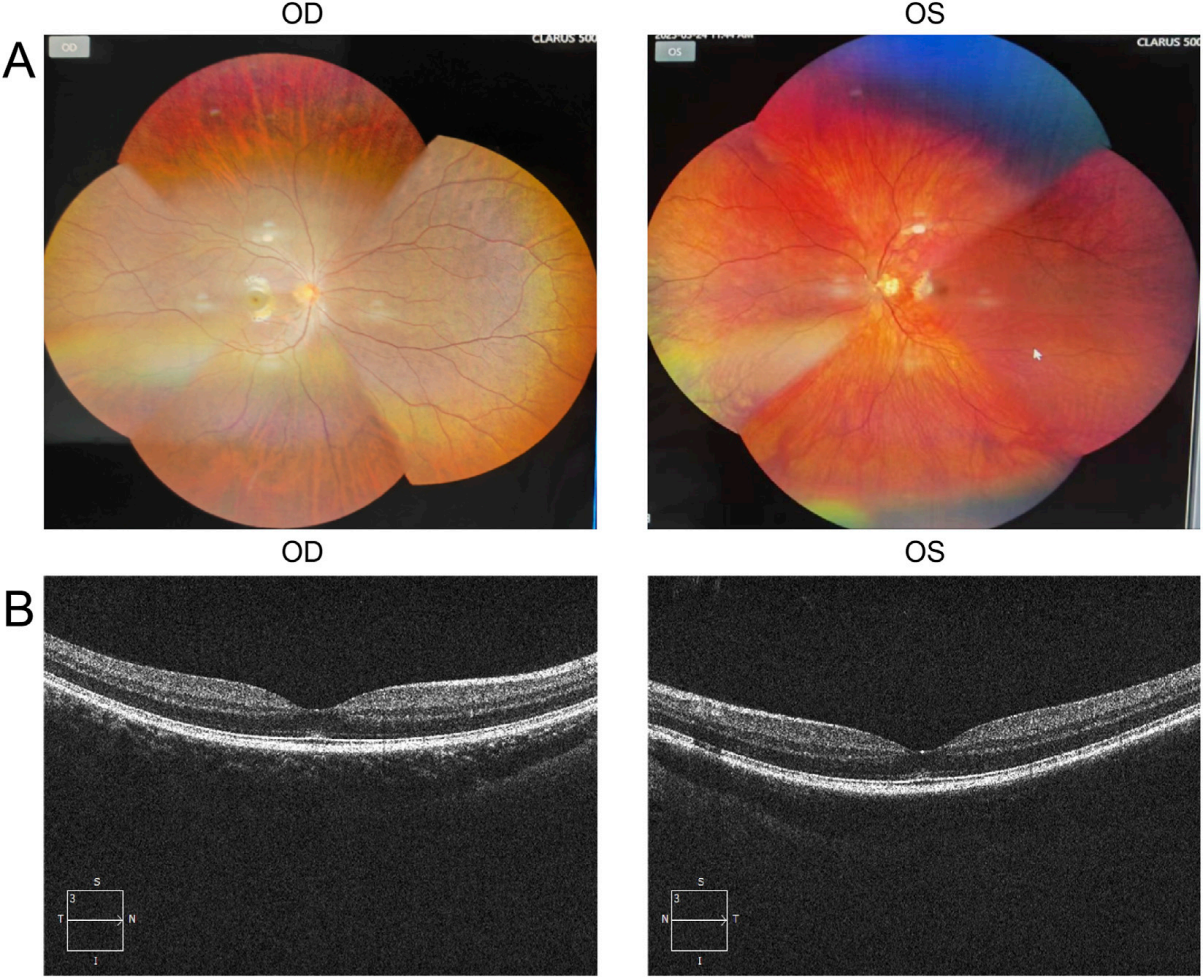
Ocular examination findings of the proband. **(A)** Color fundus photographs show an essentially normal fundus in the right eye (OD) with uniform pigment distribution, whereas the left eye (OS) exhibits markedly reduced pigmentation. **(B)** Optical coherence tomography (OCT) demonstrates intact retinal layers in the macular region of both eyes, with no evident pathological changes.

The proband is a 17-year-old female patient with congenital bilateral deafness. She had a normal pregnancy and delivery history but failed the newborn hearing screening. Her parents are non-consanguineous, and both exhibit normal hearing and no other clinical abnormalities (Figure 1A). No additional family members have reported similar symptoms. The patient’s facial features included a broad nasal root, heterochromia iridum with a blue iris in the left eye and a normally pigmented iris in the right eye, as well as thickened eyebrows on the left side (Figure 1B). The inner canthal distance (a) was 40 mm, the interpupillary distance (b) was 66 mm, and the outer canthal distance (c) was 95 mm. The W index, calculated using the standard formula, was approximately 2.10, exceeding the diagnostic threshold of 1.95. Preoperative pure-tone audiometry revealed bilateral profound sensorineural hearing loss across all frequencies (Figures 1C,D), and ABR testing failed to elicit any waveforms. Temporal bone CT and inner ear MRI showed no structural abnormalities, such as cochlear malformations. Fundus photographs demonstrated an essentially normal fundus in the right eye (OD) with uniform pigment distribution, whereas the left eye (OS) showed markedly reduced pigmentation (Figure 2A). Optical coherence tomography (OCT) demonstrated normal foveal architecture in both eyes, with intact retinal layers in the macular region and no evident pathological changes. The average retinal nerve fiber layer (RNFL) thickness in the right eye was 98 microns, with a cup-to-disc ratio of 0.37; the average RNFL thickness in the left eye was 100 microns, with a cup-to-disc ratio of 0.31 (Figure 2B). Visual function was assessed: both direct and consensual pupillary light reflexes were present; visual acuity was normal; and there was no color-vision deficiency, headache, ocular pain, restriction of extraocular movements, or visual-field defect. In addition, the patient had no musculoskeletal abnormalities, congenital aganglionic megacolon (Hirschsprung disease), or patchy cutaneous depigmentation. Based on the diagnostic criteria for Waardenburg syndrome, the patient was clinically diagnosed with WS1.At 6 years old, the patient received unilateral cochlear implantation (right side) at our institution. Intraoperative findings showed no ossicular malformations, cochlear deformities, or ossification. Additionally, there was no perilymph or cerebrospinal fluid leakage. A CI24RE (CA) cochlear implant was successfully implanted. At the 7-year postoperative follow-up, the patient showed significant improvements in hearing and speech abilities. Detailed auditory and speech evaluation scores are presented in Table 1. Before implantation, the patient had no awareness of environmental sounds and could only respond to gestures; there was no perception of environmental or speech sounds (SIR score 1; CAP, MAIS, and MUSS all 0). By the seventh year after implantation, the patient was able to communicate by telephone with familiar individuals. The CAP and SIR scores reached levels 7 and 4, respectively, while both MAIS and MUSS achieved the maximum score of 40.

**TABLE 1:** Auditory and speech evaluation scores preoperatively and at 1, 2, and 7 years after cochlear implantation.

Follow-up time	CAP score	SIR score	MAIS score	MUSS score
Pre-op	0	1	0	0
1 year post-op	2	1	10	6
2 years post-op	5	2	20	18
7 years post-op	7	4	40	40

CAP: Categories of Auditory Performance (0–7; higher = better), SIR: Speech Intelligibility Rating (1–5 scale used here; higher = better), MAIS: Meaningful Auditory Integration Scale (0–40), MUSS: Meaningful Use of Speech Scale (0–40).

### 3.2 Whole exome sequencing result analysis

We performed target gene capture and whole-exome sequencing on the proband, covering approximately 51 Mb of target regions and over 23,000 genes. The average sequencing depth was 203.59×, with 99.49% of the target region covered at a depth greater than 20×. After aligning the sequencing data with the human reference genome (GRCh37/hg19), single nucleotide variants (SNVs) and small insertions/deletions (InDels) were identified. Variants were filtered based on three main criteria: minor allele frequency of less than 0.01 in public population databases, including the 1000 Genomes Project, the NHLBI Exome Sequencing Project (ESP6500), the Exome Aggregation Consortium (ExAC), and the Genome Aggregation Database (gnomAD); localization within exons or canonical splice sites; and functional impact, specifically focusing on non-synonymous variants. Based on variant frequency, functional impact, and phenotypic association, four candidate genes—*PAX3*, *GJB3*, *DNMT1*, and *NLRP3*—were preliminarily identified. Among these, the variants in *GJB3*, *DNMT1*, and *NLRP3* were all low-frequency missense mutations lacking functional validation, phenotypic support, and familial co-segregation evidence. According to ACMG assessment, they were classified as variants of uncertain significance (VUS) and were therefore not considered pathogenic mutations (Table 2). Further analysis identified a heterozygous frameshift variant, NM_181458.4: c.788dup (p.Gln264Thrfs), in exon five of the PAX3 gene as a suspected pathogenic variant (ACMG classification: PVS1 + PM2_supporting). This variant was absent from public databases, including the Human Gene Mutation Database (HGMD), 1,000 Genomes, ESP6500, ExAC, and gnomAD, and has not been previously reported. In addition, this variant was predicted as disease-causing (probability score = 1.00) by MutationTaster.

**TABLE 2:** Summary of the four candidate gene variants identified in the proband.

Gene	cDNA change	Protein change	Zygosity	ACMG
*PAX3*(NM_181458.4)	c.788dup	p.Gln264Thrfs	Heterozygous	LP
*NLRP3*(NM_0012433.2)	c.396A>T	p.Lys132Asn	Heterozygous	VUS
*DNMT1*(NM_001130823.3)	c.3901C>T	p.Arg1301Cys	Heterozygous	VUS
*GJB3*(NM_024009.3)	c.301C>T	p.Arg101Trp	Heterozygous	VUS

LP: likely pathogenic, VUS: uncertain significance.

### 3.3 Sanger verification and effect

**FIGURE 3:**
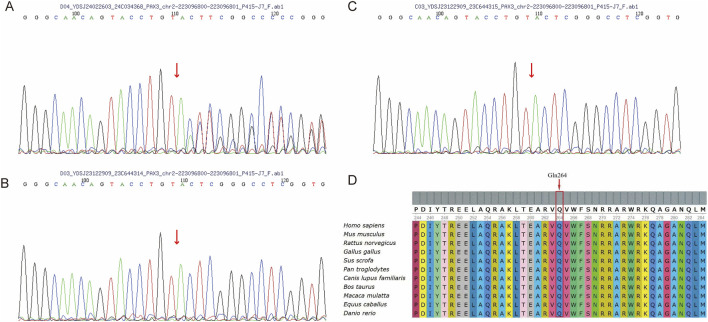
Sanger Sequencing and Conservation of the *PAX3* **(C)**788dup Variant. **(A)** Sanger sequencing of the proband (II-1) showing a heterozygous **(C)**788dup variant in *PAX3*, indicated by a red arrow. **(B,C)** Sanger sequencing of the father (B, I-1) and mother (C, I-2) shows no duplication at the **(C)**788 position in the *PAX3* gene. **(D)** Alignment of *PAX3* protein sequences from diverse species reveals that the affected residue (Gln264) is highly conserved.

**FIGURE 4:**
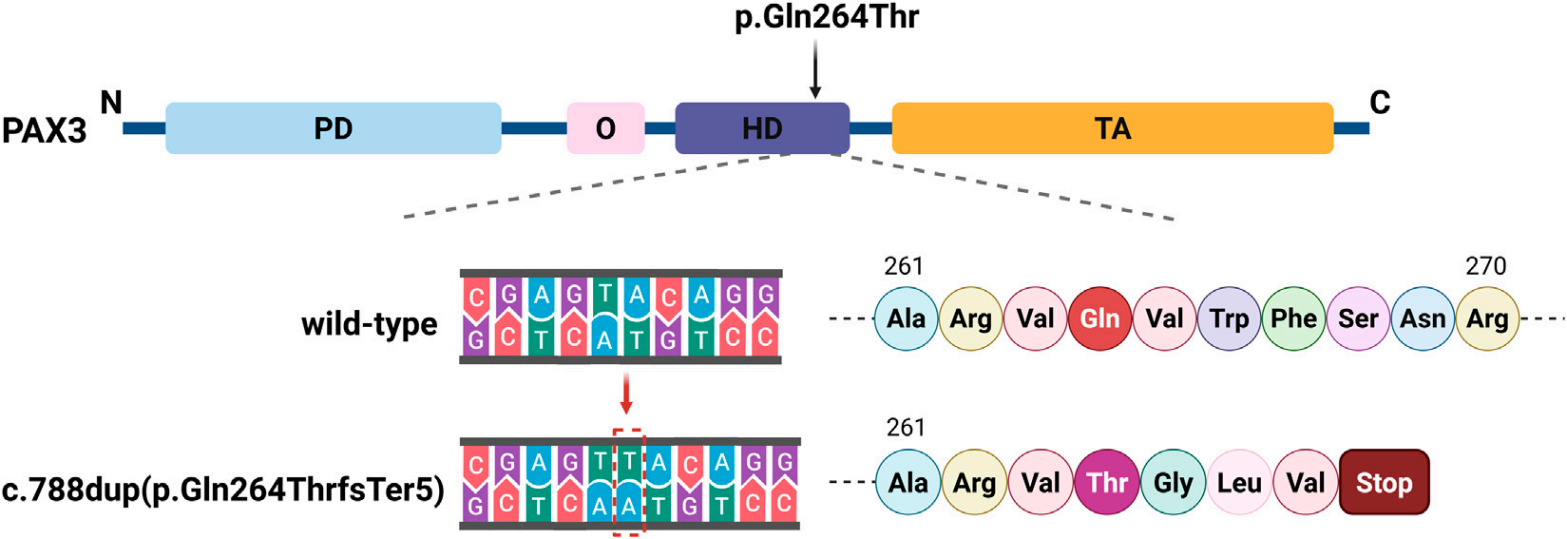
Schematic of the *PAX3* c.788dup Mutation. The *PAX3* protein includes a paired domain (PD), octapeptide (O), homeodomain (HD), and transactivation domain (TA). The c.788dup mutation causes a thymine duplication within the HD, leading to a frameshift and a premature stop codon at position 268, disrupting the downstream amino acid sequence.

The *PAX3* gene sequences obtained from the triplet samples through Sanger sequencing were compared with the reference sequence (NM_181458.4). The results showed that the proband had a clear double peak insertion pattern (heterozygous) at the c.788 site; neither parent had the insertion mutation at this site and were homozygous wild type. Therefore, this mutation is considered a *de novo* mutation (Figures 3A–C). Based on this, amino acid evolutionary conservation analysis indicated that the protein sequence in the mutated region is highly conserved during evolution, suggesting that this site is of significant importance for PAX3 protein function (Figure 3D). Further analysis revealed that this mutation is a repeated insertion-type frameshift mutation, resulting in the amino acid sequence of the PAX3 protein changing from QVWFSNRRAR at position 264 to TGLV* (* denotes a stop codon), i.e., the sequence changes begin at position 264 and terminate prematurely at position 268, leading to a truncated PAX3 protein and impaired function (Figure 4).

### 3.4 Structural modeling of the PAX3 protein

**FIGURE 5:**
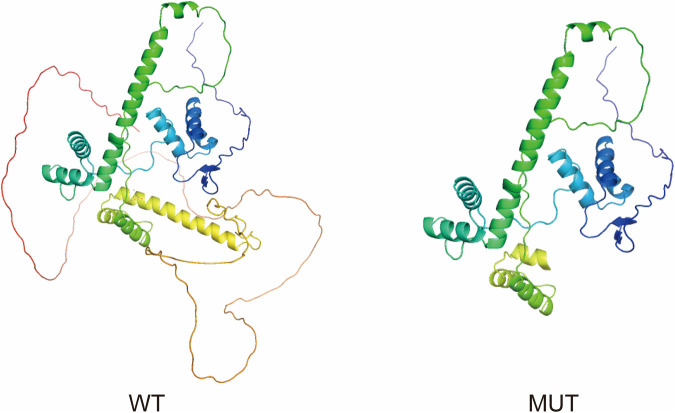
Structural comparison of wild-type and mutant *PAX3* proteins. The proteins are displayed as ribbon diagrams with a color gradient from the N-terminus (blue) to the C-terminus (red), indicating the directionality of the polypeptide chain. Ribbon diagrams show the wild-type (WT) protein with intact secondary structures, especially at the C-terminus. The mutant (MUT) protein, affected by a frameshift, displays a truncated C-terminus and loss of several α-helices, potentially impairing structural stability and function.

To investigate how this variant might affect the structure of PAX3, structural predictions were conducted using AlphaFold to generate three-dimensional models for both the normal and altered PAX3 proteins. The modeling results showed that the wild-type protein (WT) structure remained intact, while the mutant protein (MUT) exhibited significant conformational changes at the C-terminus, resulting in the loss of the functional domain at the C-terminus of the PAX3 protein. We used PyMOL software to perform visualization analysis of the wild-type and mutant protein structures. The results indicated that the mutation significantly altered the spatial conformation of the PAX3 protein’s C-terminal segment, potentially disrupting its normal DNA binding and downstream gene regulatory functions (Figure 5).

## 4 Discussion

In this study, we identified a novel *PAX3* mutation (c.788dup) in a Yugur family with WS1 and congenital deafness. This frameshift variant introduces a premature stop codon, producing a truncated protein lacking the C-terminal functional domain. Structural modeling and bioinformatic predictions support its pathogenicity. Combined with clinical features and segregation analysis, the c.788dup mutation is likely the molecular cause of WS1 in this family.

*PAX3* gene encodes a critical transcription factor that is highly expressed in neural crest cells during early embryogenesis, where it promotes their differentiation into specific tissues and cell types. Under physiological conditions, *PAX3* activates downstream genes, including melanocyte-inducing transcription factor (MITF), which in turn regulates the transcription of key enzymes involved in melanin biosynthesis, playing a pivotal role in melanocyte differentiation. The loss of melanocytes causes pigmentation abnormalities in the skin, hair, and eyes, along with hearing defects due to cochlear dysfunction, resulting in typical WS symptoms (Li et al., 2024). Niu et al. also identified a novel *PAX3* variant (c.117C>A; p.Asn39Lys) in a four-generation Chinese family with Waardenburg syndrome type 1 (WS1). They found that this variant impaired the function of wild-type PAX3, preventing activation of the MITF promoter and ultimately leading to the WS clinical phenotype (Niu et al., 2021).

**FIGURE 6:**
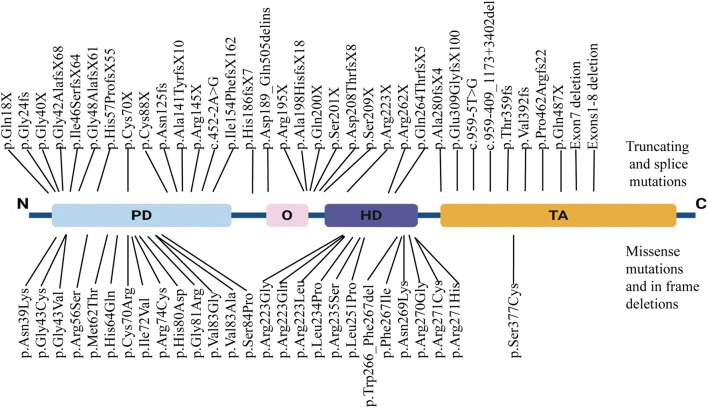
Distribution of PAX3 variants in Chinese patients with Waardenburg syndrome. Truncating and splice-site variants (nonsense, frameshift, and canonical splice-site variants) are shown above the schematic, whereas missense variants and in-frame indels are shown below. Variants span all domains, with notable clustering in the paired domain (PD) and homeodomain (HD).

To date, a total of 226 pathogenic mutations have been identified in the *PAX3* gene, including missense, nonsense, small insertions, small deletions, and splicing site mutations (https://www.hgmd.cf.ac.uk/ac/gene.php?gene=PAX3). Missense/nonsense mutations account for approximately 51% of *PAX3* variants (n = 115), while small insertions represent about 6% (n = 14). Most mutations cluster between exons two and 6, affecting the PD and HD. Studies have shown that approximately 80% of WS patients have heterozygous mutations in the *PAX3* gene. We have systematically summarized the *PAX3* mutations reported in Chinese WS patients (Table 3). Our review found that over 60 different *PAX3* mutations have been reported among Chinese WS patients, with truncating mutations accounting for about 48%, followed by missense mutations. In Chinese patients with WS, *PAX3* variants are mainly concentrated in the PD and HD; the detailed distribution is shown in Figure 6. Several variants have been reported repeatedly and may represent hotspots in this population, including c.667C>T, c.117C>A, c.123del, c.127G>T, and c.452–2A>G. WS is inherited in an autosomal dominant manner. While most PAX3 variants in WS are inherited from a parent, multiple studies have also reported *de novo* mutations. In a cohort of 90 WS probands, Wang et al. identified *PAX3* variants in 13 individuals (14.4%; 13/90), three of which were *de novo* (c.668C>T, c.752T>C, and c.922dupG), accounting for 23.1% (3/13) of *PAX3*-positive cases (Wang et al., 2022). In addition, *de novo PAX3* variants reported in Chinese WS patients include c.166C>A, c.433C>T, c.456_459dupTTCC, c.592delG, c.626_627delCT, c.667C>G, c.668G>T, c.795_800delCTGGTT, c.1459C > T; none were detected in either parent, and most were truncating or missense variants. In our study, the *PAX3* c.788dup variant was likewise absent in both parents, confirming it to be *de novo*. This variant occurs in exon five and alters the PAX3 amino acid sequence beginning at residue 264 (p.Gln264Thrfs); this region lies within the highly conserved homeodomain, which mediates DNA binding. According to MutationTaster, the variant is predicted to cause a frameshift, resulting in a premature termination codon (PTC) and likely triggering nonsense-mediated mRNA decay (NMD). Additionally, the mutation may impair both protein domain function and splicing regulation, ultimately leading to loss of PAX3 function.

**TABLE 3:** The reported PAX3 mutations in Chinese patients with Waardenburg syndrome.

cDNA change	Protein change	Mutation type	Inheritance	References
c.52C>T	p.Gln18X	Nonsense	N.R.	Lee et al. (2022)
c.72delG	p.Gly24fs	Frameshift	N.R.	Hao et al. (2016)
c.117 C > A	p. Asn39Lys	Missense	Familial	Niu et al. (2021)
c.118C>T	p.Gln40X	Nonsense	N.R.	Hao et al. (2016)
c.123del	p.Gly42AlafsX68	Frameshift	Familial	Zhang et al. (2021)
c.127G > T	p.Gly43Cys	Missense	Familial	Guo et al. (2021)
c.128G > C	p.Gly43Val	Missense	N.R.	Hao et al. (2016)
c.136delA	p.Ile46SerfsX64	Frameshift	N.R.	Huang et al. (2022a)
c.143delG	p.Gly48AlafsX61	Frameshift	Familial	Wang et al. (2022)
c.166C>A	p.Arg56Ser	Missense	*De novo*	Poon et al. (2025)
c. [169_170insC; 172_174delAAG]	p.His57ProfsX55	Frameshift	Familial	Xiao et al. (2016)
c.185T > C	p.Met62Thr	Missense	N.R.	Hao et al. (2016)
c.192C>A	p.His64Gln	Missense	N.R.	Lee et al. (2022)
c.208 T > C	p.Cys70Arg	Missense	Familial	Xiao et al. (2023)
c.210C > A	p.Cys70X	Nonsense	Familial	Wang et al. (2022)
c.214A > G	p.Ile72Val	Missense	N.R.	Li et al. (2022)
c.220C > T	p.Arg74Cys	Missense	Familial	Liu et al. (2020)
c.238C>G	p.His80Asp	Missense	Familial	Chen et al. (2010)
c.241G > C	p.Gly81Arg	Missense	Familial	Wang et al. (2022)
c.248 T>C	p.Val83Ala	Missense	Familial	Poon et al. (2025)
c.248T>G	p.Val83Gly	Missense	N.R.	Sun et al. (2016)
c.250T>C	p.Ser84Pro	Missense	Familial	Poon et al. (2025)
c.264delC	p.Cys88X	Nonsense	Familial	Wang et al. (2022)
c.372-373delGA	p.Asn125fs	Frameshift	Familial	Yu et al. (2020)
c.420-424delCGCGGinsTTAC	p.Ala141TyrfsX10	Frameshift	Familial	Ma et al. (2019)
c.433C>T	p.Arg145X	Nonsense	*De novo*	Poon et al. (2025)
c.452–2A > G (x2)	N.A.	Splicing	Familial	Li et al. (2022)
c.456_459dupTTCC	p.Ile154PhefsX162	Frameshift	*De novo*	Wang et al. (2010)
c.556delC	p.His186ThrfsX7	Frameshift	N.R.	Chen et al. (2010)
c.567_586 + 17del	p.Asp189_Gln505delins	Splicing	Familial	Wang et al. (2010)
c.583C>T	p.Arg195X	Nonsense	Familial	Wang et al. (2017)
c.592delG	p.Ala198HisfsX18	Frameshift	*De novo*	Chen et al. (2018)
c.598C>T	p.Gln200X	Nonsense	Familial	Chen et al. (2017)
c.602C>G	p.Ser201X	Nonsense	Familial	Li et al. (2021)
c.622delG	p.Asp208ThrfsX8	Frameshift	N.R.	Poon et al. (2025)
c.626_627delCT	p.Ser209X	Nonsense	*De novo*	Yang et al. (2007)
c.667C>G	p.Arg223Gly	Missense	*De novo*	Poon et al. (2025)
c.667C>T	p.Arg223X	Nonsense	Familial	Wang et al. (2023)
c.668G>T	p.Arg223Leu	Missense	*De novo*	Huang et al. (2022a)
c.668C > T	p.Arg223Gln	Missense	*De novo*	Wang et al. (2022)
c.701T > C	p.Leu234Pro	Missense	Familial	Qin et al. (2006)
c.703C > A	p.Arg235Ser	Missense	Familial	Wang et al. (2022)
c.752T > C	p.Leu251Pro	Missense	*De novo*	Wang et al. (2022)
c.784C>T	p.Arg262X	Nonsense	Familial	Sun et al. (2016)
c.788dup	p.Gln264ThrfsX5	Frameshift	*De novo*	This study
c.795_800delCTGGTT	p.Trp266_Phe267del	In-frame deletion	*De novo*	Wang et al. (2010)
c.799T>A	p.Phe267Ile	Missense	Familial	Wang et al. (2010)
c.807C>A	p.Asn269Lys	Missense	N.R.	Lee et al. (2022)
c.808C>G	p.Arg270Gly	Missense	Familial	Niu et al. (2018)
c.811C>T	p.Arg271Cys	Missense	Familial	Lin et al. (2023)
c.812G > A	p.Arg271His	Missense	Familial	Chen et al. (2010)
c.838delG	p.Ala280fsX4	Frameshift	Familial	Li et al. (2022)
c.922dupG	p.Glu309GlyfsX100	Frameshift	*De novo*	Wang et al. (2022)
c.959–5T>G	N.A.	Splicing	Familial	Hu et al. (2021)
c.959-409_1173 + 3402del	N.A.	Deletion	Familial	Zhang et al. (2021)
c.1076_1077del	p.Thr359fs	Frameshift	Familial	Li et al. (2020)
c.1174–2A>T	p.Val392fs	Splicing	Familial	Sun et al. (2016)
c.1130C>G	p.Ser377Cys	Missense	N.R.	Lee et al. (2022)
c.1385_1386delCT	p.Pro462Argfs22	Frameshift	Familial	Bai et al. (2016)
c.1459C > T	p.Gln487X	Nonsense	*De novo*	Zhang et al. (2021)
Exon7 deletion	N.A.	CNV	Familial	Bai et al. (2016)
Exon1-8 deletion	N.A.	CNV	Familial	Yang et al. (2023)

N.R. = not recorded; N.A. , not applicable; CNV = Copy-number variants.

Several scientific reports confirm that mutations in the HD region of PAX3 alter its spatial conformation, disrupting its nuclear localization and chromatin binding capacity, thereby impairing its function as a transcription factor. Corry et al. reported that HD mutations significantly compromise the dynamic localization and nuclear anchoring of PAX3, leading to aberrant regulation of downstream genes (Corry et al., 2008). Wu et al. further observed that HD-mutated PAX3 variants fail to bind chromosomes during mitosis, thereby affecting cell proliferation and differentiation (Wu et al., 2015). Additionally, HD mutations in PAX3 have been shown to prevent the activation of the MITF promoter, resulting in impaired melanocyte differentiation and survival in WS1 patients (Watanabe et al., 1998). Therefore, this mutation is likely to reduce PAX3 protein levels through mechanisms such as NMD, leading to haploinsufficiency and resulting in pathogenicity (Huang L. et al., 2022).

It is noteworthy that the *PAX3* mutation identified in this study (c.788dup, p.Gln264Thrfs) is a frameshift variant, whereas the literature also reports a missense mutation at the same nucleotide position, *PAX3* (c.788T>A, p.Val263Gly), both of which are associated with the typical WS1 phenotype (Hazan et al., 2013). This observation suggests that this site is critical for maintaining the structure and transcriptional activity of *PAX3*. Moreover, several studies have shown that even identical mutations at the same site can exhibit markedly different clinical phenotypes in Waardenburg syndrome. Guo et al. identified a novel missense mutation in exon two of *PAX3* (c.127G>A) in the proband of a Han Chinese WS1 family with hearing loss; the proband had unilateral moderate hearing loss, whereas his mother carrying the same mutation exhibited bilateral profound deafness (Guo et al., 2021). Ma et al. reported a WS1 patient diagnosed with a new *PAX3* mutation (c.420-424delCGCGGinsTTAC) who had bilateral congenital sensorineural hearing loss, yet the proband’s sister carrying the same mutation had normal hearing (Ma et al., 2019). Such variability may be attributed to the combined influence of epigenetic modifications and environmental factors on the penetrance and expressivity of pathogenic mutations.

Many studies have sought to clarify genotype–phenotype correlations in WS. Li et al. conducted genotype–phenotype assessments in eight Chinese WS families and identified several novel *PAX3* variants (c.838delG, c.452–2A>G, and c.214A>G). They also observed that cases carrying *PAX3* mutations often exhibited unilateral hearing loss and marked clinical variability (Li et al., 2022). Wang et al. reported the clinical and genetic features of 90 Chinese WS probands, among whom 13 (14.4%) harbored *PAX3* mutations; that study further found that *PAX3* variants were more frequently associated with asymmetric hearing loss and moderate hearing impairment (Wang et al., 2022). By enrolling 30 genetically confirmed Chinese WS patients and comparing them with other cohorts in the literature, Poon et al. identified six novel *PAX3* variants (c.250T>C, c.166C>A, c.248T>C, c.667C>G, c.433C>T, and c.622delG). In that cohort, 22% of WS patients with *PAX3* variants presented with asymmetric or unilateral hearing loss, and the prevalence of hearing loss reached 43%, findings similar to those of Wang et al. (Poon et al., 2025).

Currently, there is no cure for WS, and clinical management mainly relies on supportive measures such as hearing aids and cochlear implants to improve hearing (Huang S. et al., 2022). In this case, the patient achieved significant auditory and speech recovery after cochlear implantation and rehabilitation. Three years post-surgery, she successfully transferred to a regular school and is now able to communicate by phone with familiar individuals (CAP level 7), underscoring the importance of early genetic diagnosis and timely intervention in WS1 with severe hearing loss. WS involves multiple organ systems and therefore requires comprehensive, multidisciplinary management. In addition to hearing, attention should be given to patients’ optometric/visual needs, photoprotection, cosmetic concerns, and psychological wellbeing, with appropriate education and interventions provided during follow-up. During follow-up, we offered psychological support and genetic counseling to the patient and family to enhance their understanding of the condition. Moreover, for cosmetic concerns arising from differences in hair or iris pigmentation, we advised options such as appropriate hair dyeing or tinted contact lenses. For concerns about the visibility of the external cochlear-implant sound processor, we suggested physical concealment using hairstyles or accessories. With the development of gene therapy and other technologies, precise treatments targeting such loss-of-function mutations may become feasible in the future. For example, Yao et al. successfully corrected the pathogenic point mutation (*MITF* c.740T>C) using CRISPR-Cas9-mediated gene editing, restoring vision and hearing loss in a WS pig model (Yao et al., 2021). Additionally, Li et al. developed inner ear organoid models from induced pluripotent stem cells (iPSCs) derived from WS1 patients carrying the *PAX3* c.214A>G mutation and from healthy controls. Using CRISPR/Cas9, they corrected the *PAX3* mutation in patient-derived iPSCs. In the corrected organoids, they observed restoration of WNT1/β-catenin signaling, reduced apoptosis, increased organoid size, and significant reversal of cochlear developmental defects associated with WS1 (Li et al., 2024).

In summary, the novel heterozygous *PAX3* mutation identified in this study is the likely genetic cause of WS1 in this family, expanding the known mutation spectrum and contributing to research in minority populations. Future efforts should focus on functional validation, animal models, and gene-editing strategies. Multi-omics approaches may clarify phenotypic variability. Clinically, early genetic screening, neonatal hearing tests, and counseling are essential. Advances in gene therapy, such as CRISPR/Cas9, hold promise for precise, targeted treatment of WS.

## Data Availability

The original contributions presented in the study are publicly available. This data can be found here: https://www.ncbi.nlm.nih.gov/bioproject/PRJNA1345158. BioProject accession: PRJNA1345158; BioSample accession: SAMN52662672.
